# Neurosurgery-Led Digital Emergency Referral System in Khyber Pakhtunkhwa, Pakistan: Protocol for a Mixed Methods Implementation Study

**DOI:** 10.2196/90331

**Published:** 2026-07-17

**Authors:** Muhammad Nawaz Khan, Tehsina Ali, Muhammad Sohaib Khan, Syed Shayan Shah, Bilal Bashir, Ali Talha, Adnan Khan, Syed Jawad Ahmad, Muhammad Junaid

**Affiliations:** 1 Department of Neurosurgery Lady Reading Hospital Peshawar, Khyber Pakhtunkhwa Pakistan; 2 Department of Gynaecology Mian Rashid Hussain Shaheed Memorial Hospital Nowshehra, Khyber Pukhtunkhwa Pakistan; 3 Department of IT Lady Reading Hospital Peshawar, Khyber Pakhtunkhwa Pakistan; 4 Department of Pathology Lady Reading Hospital Peshawar, Khyber Pakhtunkhwa Pakistan; 5 Department Of Neurosergery Combined Military Hospital Rawalpindi, Punjab Pakistan

**Keywords:** referral and consultation, telemedicine, emergency medical services, health information systems, neurosurgery

## Abstract

**Background:**

Emergency referral systems in low- and middle-income countries (LMICs) are characterized by systemic inefficiencies, including prolonged transfer delays, interfacility miscommunication, and inadequate receiving-end preparation. In Khyber Pakhtunkhwa (KP), a province of Pakistan with a population exceeding 40 million, referral pathways remain predominantly paper-based and operationally fragmented. Tertiary medical institutions, including Lady Reading Hospital (LRH), Khyber Teaching Hospital (KTH), and Hayatabad Medical Complex (HMC), sustain a disproportionate burden of medically unnecessary referrals from peripheral facilities, culminating in institutional overcrowding, resource depletion, and attenuation of specialist neurosurgical and emergency care delivery.

**Objective:**

This study aims to design, implement, and evaluate a neurosurgery-led digital emergency referral platform—the KP Medical Teaching Institution (MTI) Referral Application—across primary, secondary, and tertiary tiers of health care delivery in KP, with the primary intent of reducing unnecessary referral rates, abbreviating referral response intervals, optimizing inpatient bed utilization, and strengthening bidirectional inter-facility communication.

**Methods:**

A convergent mixed methods design will be employed, incorporating a quantitative prospective cohort study alongside a qualitative exploratory component. The quantitative arm will adopt a census-based methodology, enumerating all emergency referrals processed through the digital platform over a 6-month pilot period commencing June 2026, with preimplementation historical referral data serving as the comparator. An estimated 5000-7000 referral episodes are anticipated. Primary outcomes include reduction in unnecessary referral rates and referral-to-response time. Secondary outcomes encompass inpatient bed occupancy rates, unanswered referral proportions, case acceptance and declination rates, remotely managed cases precluding physical transfer, patient mortality indices where ascertainable, and health care provider satisfaction scores. Statistical analysis will be performed using IBM SPSS, incorporating descriptive statistics, chi-square, or Fisher exact tests, paired comparisons, and multivariate logistic regression. The qualitative component will comprise structured surveys, semistructured interviews, and focus group discussions among purposively sampled health care professionals and patients, with thematic analysis conducted independently by 2 coders and findings integrated via a convergent mixed methods framework.

**Results:**

System development, stakeholder engagement, user-centered iterative design, beta testing, and platform validation have been completed. Participant recruitment is pending pilot deployment in June 2026. Data collection is projected to conclude by November 2026, followed by analysis from December 2026 through February 2027. Peer-reviewed dissemination is anticipated in spring 2027. No external funding was received.

**Conclusions:**

This protocol outlines an integrated, multimodal assessment of a neurosurgery-based digital emergency referral system in a resource-constrained low- to middle-income country. In case the expected gains in referral efficiency and communication are realized, the results can be valuable evidence to justify the wider implementation of digital referral platforms in KP and other similar environments.

**International Registered Report Identifier (IRRID):**

PRR1-10.2196/90331

## Introduction

Digital health has become a key facilitator of health care provision in low- and middle-income countries (LMICs), where resource scarcity, geographic challenges, and systemic inefficiencies restrict access to timely care [1]. Mobile health technologies serve as health system–strengthening tools supporting health care professional-to-health care professional communication, clinical decision-making, and coordination of patient care across levels of the health system [2]. Electronic referral (e-referral) systems specifically have been receiving growing interest as scalable solutions to delays and miscommunication that are inherent in paper-based referral pathways [3].

Emergency referral systems in Pakistan are still largely paper based and very fragmented. Timely and effective emergency referrals are especially critical for neurosurgical conditions—including traumatic brain injury, spinal trauma, and acute stroke—where delays of even a few hours can result in permanent disability or death [4]. The health care system is overstretched in Khyber Pakhtunkhwa (KP), a Pakistani province with a population of more than 40 million. The area is typified by a predominantly rural population, a history of conflict-related displacement, and a lack of health care infrastructure in peripheral regions [5].

High numbers of preventable referrals to tertiary care hospitals are regularly received by Lady Reading Hospital (LRH), Khyber Teaching Hospital (KTH), and Hayatabad Medical Complex (HMC). These unnecessary transfers are made without checking bed availability or specialist capacity, creating unnecessary patient transfers, increasing health care expenses, and exacerbating overcrowding in tertiary facilities [6]. This is further complicated by self-referred patients. At the same time, district-level facilities are underused due to the lack of resources and staffing, which leads to an imbalance paradox within a single provincial health system.

The fast growth of mobile and internet connectivity in Pakistan offers a favorable setting to digital health interventions. Mobile phone penetration grew to over 80% in 2022 as compared to less than 10% in 2000, with over 190 million cellular connections and over 124 million broadband subscribers registered in 2023 [7]. This online base offers a significant prospect to implement mobile-based referral systems even in rural and underserved areas of KP.

Worldwide, digital referral systems have shown promising results in LMICs. The Mobile Obstetric Referral Emergency System in Liberia enhanced communication between health care providers and facilitated timely obstetric referrals in rural areas [8]. In Saudi Arabia, the Saudi Medical Appointments and Referrals Centre improved the coordination of patient transfers at a national level, although system integration was problematic [9]. More recently, web-based acute neurosurgical referral systems have shown better specialist response time and quality of referrals in high-income environments, highlighting the translational potential to LMICs [10]. Evidence from Indonesia demonstrates the feasibility of structured digital referral pathways in resource-constrained settings [11], whereas systematic reviews of e-referral implementations confirm their potential to reduce delays and improve care coordination across health care levels [12].

It has been shown that the key to the successful implementation of digital health systems is the involvement of stakeholders, the ability to integrate with the current clinical processes, the provision of appropriate user training, and the maintenance of monitoring and evaluation [13]. The lack of consideration of these factors has led to low adoption and low sustainability of previous LMIC implementations. The current intervention development is informed by the principles of user-centered design and implementation science frameworks, namely, the Consolidated Framework for Implementation Research and the World Health Organization *Digital implementation investment guide*, which focus on system usability, flexibility, and continuous improvement through close collaboration with end users [14].

Although there is an increasing body of evidence on the topic of digital health in LMICs, digital referral systems led by neurosurgery and specifically designed to meet the needs of complex provincial health systems in Pakistan are not represented in the literature. This study fills that gap by developing, deploying, and assessing the KP MTI Referral Application, a mobile- and web-based emergency referral application that was developed specifically to support the health care infrastructure of KP, through a rigorous mixed methods implementation process. We assume that the introduction of such a system will help decrease the number of unnecessary transfers of patients, decrease the time of referral initiation to response, optimize bed use, and increase 2-way communication between health care institutions of various levels of care.

## Methods

### Study Design

This is a mixed methods implementation study with a prospective cohort design for the quantitative component and a qualitative exploratory component. The quantitative component captures all emergency referrals recorded through the digital platform during a defined 6-month pilot period using preimplementation historical referral data from participating institutions for pretest-posttest comparison. The qualitative component examines health care provider and patient experiences through surveys, semistructured interviews, and focus group discussions. Quantitative and qualitative findings will be integrated using a convergent mixed methods framework.

### Study Setting

The research will be carried out at various levels of the Pakistani public health care system in KP. Primary care is provided by tehsil (subdistrict-level) hospitals and basic health units, secondary care is provided by district headquarters hospitals, and tertiary care is provided by Medical Teaching Institutions (MTIs). The 3 large tertiary care MTIs involved in this study are LRH, KTH, and HMC, all in the provincial capital, Peshawar.

### Platform Development and Validation

The KP MTI Referral Application was developed through a user-centered design approach informed by continuous stakeholder engagement and iterative refinement. Structured requirement-gathering sessions were conducted with neurosurgeons, emergency physicians, medical officers, nurses, bed managers, hospital administrators, and IT personnel from participating institutions to ensure alignment with existing referral workflows and operational requirements.

A beta version of the platform was deployed in a controlled testing environment at LRH. Beta testing involved volunteer health care providers, including physicians and bed managers, who participated in simulated trauma referral scenarios. Testing evaluated successful referral submission, transmission of clinical information and images, notification delivery, specialist response receipt, referral tracking functionality, and overall usability. End-to-end workflow testing confirmed that referrals could be submitted successfully, notifications were delivered appropriately, specialist responses were received and tracked, and referral progress could be monitored in real time.

Feedback obtained during beta testing informed iterative platform refinements. Major modifications included simplification of the referral form interface, optimization of image-sharing workflows, and enhancement of the notification system through automated reminder alerts repeated every 10 minutes until a specialist response was documented. Validation testing was subsequently conducted against predefined functional and clinical requirements before pilot deployment. Formal training materials and implementation support resources were also developed for frontline users prior to rollout.

### Intervention

**Figure 1:**
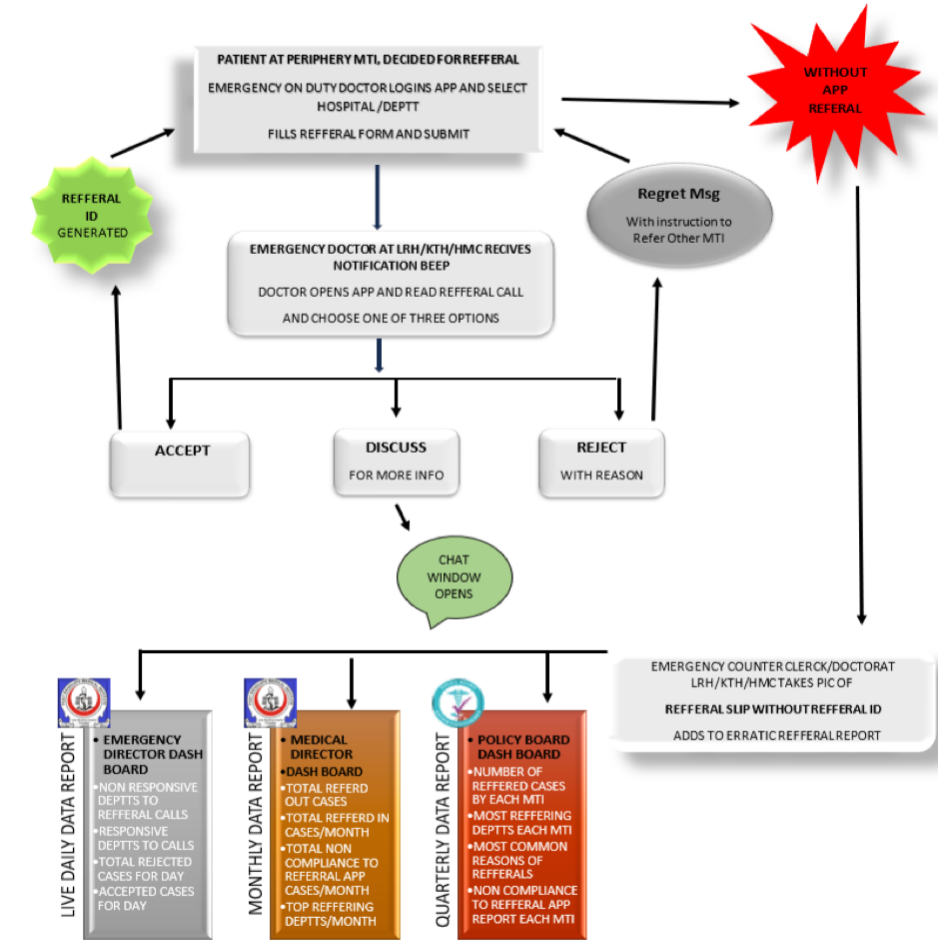
Flowchart of the digital emergency referral process using the proposed referral application. Patients requiring referral from peripheral Medical Teaching Institutions (MTIs) are electronically referred to tertiary care hospitals, where the on-duty emergency physician reviews the referral and either accepts, discusses, or rejects the request. Accepted referrals generate a unique referral identification (ID), while rejected referrals include a documented reason and instructions for onward referral when appropriate. The system also provides real-time communication through a chat function and generates dashboards for monitoring referral activity and compliance. App: application; Deptt: department; HMC: Hayatabad Medical Complex; KTH: Khyber Teaching Hospital; LRH: Lady Reading Hospital; Msg: message.

**Figure 2:**
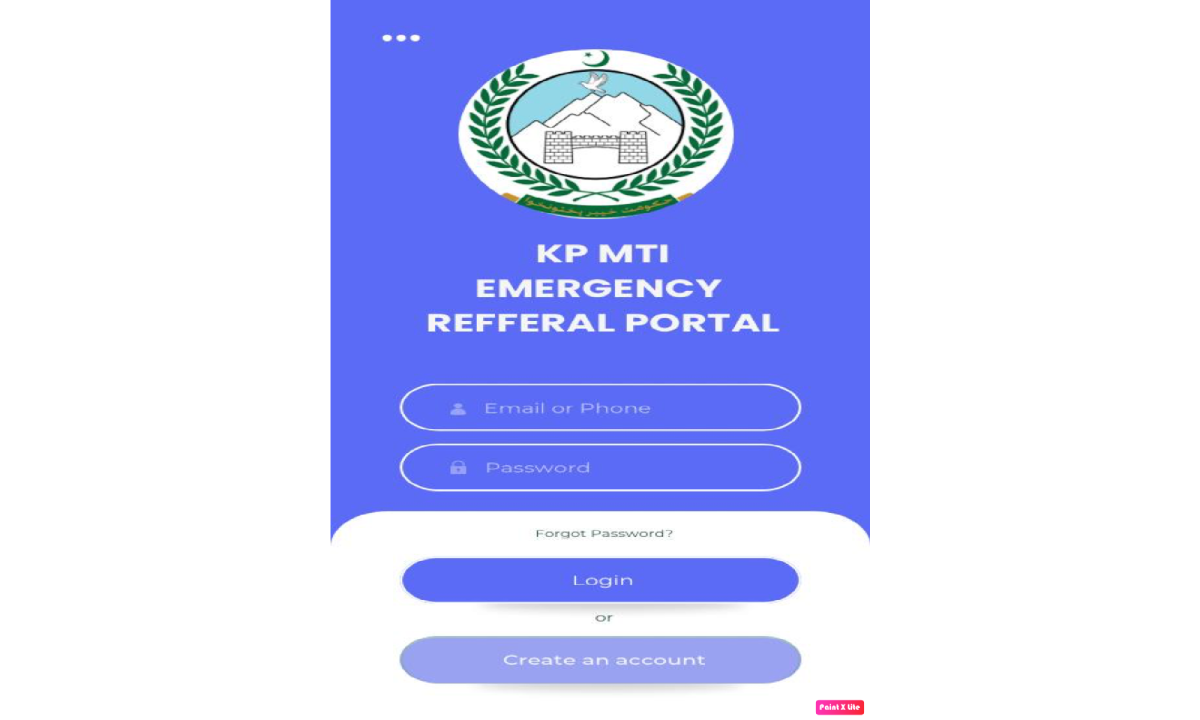
Login interface of the KP MTI Emergency Referral Portal mobile app. The login screen allows authorized health care providers to securely access the referral system using their registered email address or phone number and password. The interface also includes options for password recovery and new account registration. KP: Khyber Pakhtunkhwa; MTI: Medical Teaching Institution.

**Figure 3:**
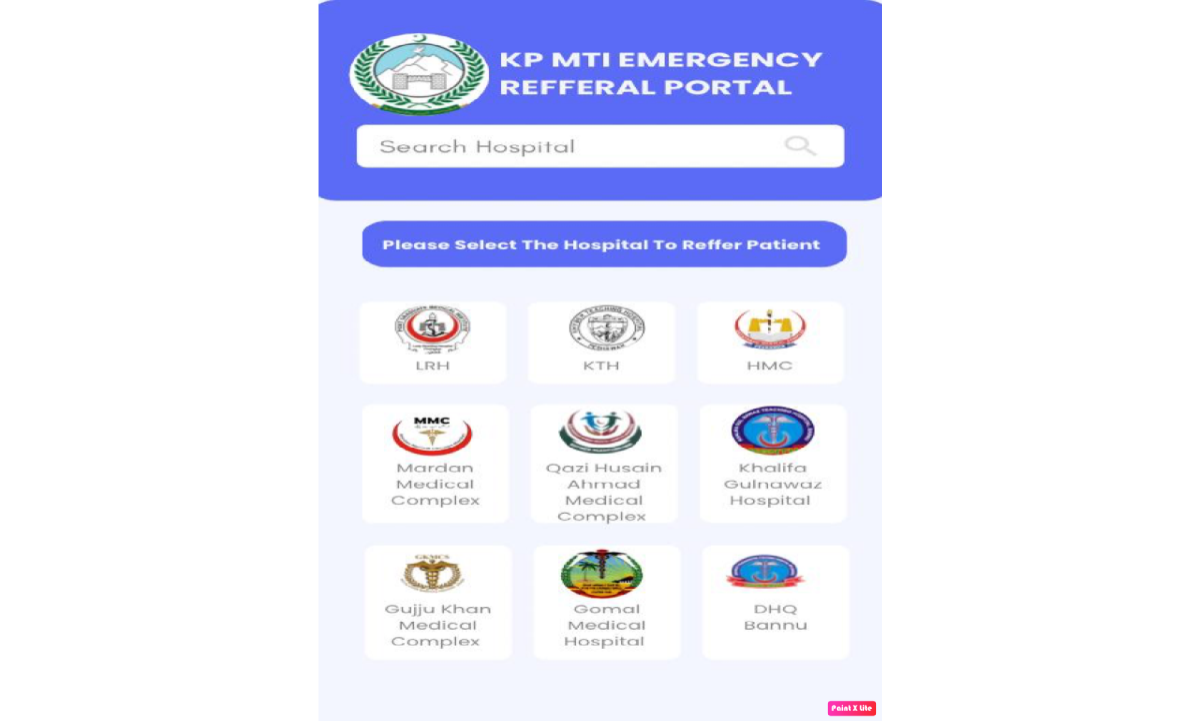
Hospital selection interface of the KP MTI Emergency Referral Portal mobile app. After logging in, the referring physician can search for and select the destination hospital from the list of participating health care facilities before initiating an emergency referral. The interface includes tertiary care and affiliated Medical Teaching Institutions (MTIs) within Khyber Pakhtunkhwa (KP). DHQ: District Headquarters Hospital; HMC: Hayatabad Medical Complex; KTH: Khyber Teaching Hospital; LRH: Lady Reading Hospital.

The intervention is a digital emergency referral platform, which encompasses a mobile app and web-based application—the KP MTI Referral Application. The platform offers a standardized electronic referral form, bed availability in real time, unique referral identifiers, and triage by urgency (emergency, urgent, and routine; Table 1). The platform has a 2-way communication feature that enables receiving specialists to give clinical management advice to referring health care professionals without the latter necessarily transferring the patient. Automated notifications are set based on the urgency category, with time-sensitive alerts on emergency and urgent referrals and regular alerts on routine referrals. This urgency-stratified method does not cause alarm fatigue linked to universal reminders. Figure 1 illustrates the referral process workflow. Figures 2 and 3 show mobile app interfaces.

**Table 1:** Platform components and their purpose.

Platform component	Function	User group
Standardized electronic referral form	Captures demographics, clinical summary, diagnosis, and urgency category	Referring physicians, nurses, and medical officers
Image and document upload	Transmits clinical photos and imaging for remote specialist review	Referring health care professionals
Real-time bed availability display	Enables informed referral destination selection	All referring health care professionals
Urgency triage classification	Stratifies referrals as emergency, urgent, or routine to prioritize responses	Referring health care professionals and triage nurses
Unique referral identifier	Enables end-to-end tracking of each referral from initiation to outcome	All users and administrators
Bidirectional communication function	Allows specialists to provide management advice without requiring transfer	Receiving specialists and referring health care professionals
Urgency-based automated notifications	Alerts for emergency and urgent referrals and standard notifications for routine referrals	Receiving specialists and bed managers

### Participants and Recruitment

The research will engage health care professionals such as emergency physicians, consultants, medical officers, nurses, bed managers, and administrative personnel who have access to referral dashboards to monitor and coordinate. The patients or their legal attendants who undergo the referral process within the study period will also be invited to participate.

### Eligibility Criteria

In the quantitative component, all the emergency referral records created via the digital platform within the 6-month study period will be considered. Records will be omitted when they are not complete, marked as duplicates, or created in the process of system testing. In the qualitative component, health care providers will be eligible if they have made at least one referral through the digital platform. Patients or attendants will be included if they have been referred to a hospital via the platform. Those who do not give consent will be excluded.

### Sample Size and Sampling

The quantitative part will use a census-based cohort design: all the referral records created via the platform over the 6-month pilot will be considered. According to the administrative referral logs of LRH, KTH, and HMC, it is estimated that 5000 to 7000 emergency referrals will be made. As the sample size of a census-based implementation study cannot be calculated using formal power, the rationale behind the sample size is the expected volumes of institutional referrals. For the qualitative component, health care providers will be selected using stratified purposive sampling to ensure representation across facility levels (primary, secondary, and tertiary), clinical roles (physicians, nurses, and administrators), and geographic locations (urban and rural). Recruitment will continue until thematic saturation is reached, with an anticipated sample of 20 to 30 individual interviews and 4 to 6 focus group discussions involving 6 to 8 participants each. Patients and attendants will be recruited using convenience sampling at the time of referral completion.

### Outcomes

Primary outcomes are change in the proportion of unnecessary referrals (defined as referrals where the receiving specialist determines that the patient could have been managed at the referring facility without transfer) and change in referral initiation–to-response time. Secondary outcomes are change in bed use, number of unanswered referral requests, referral acceptance and decline rates, number of cases managed through remote advice without transfer, patient mortality among emergency referrals where feasible, referral-related costs where feasible, patient and health care professional satisfaction, and platform usability (Table 2).

**Table 2:** Outcome definitions, data sources, and analysis plan.

Outcome	Definition	Data source	Analysis plan
Change in unnecessary referral rate	Referrals deemed manageable at the referring facility without transfer	Platform records+specialist response field	Chi-square test and multivariable logistic regression
Change in referral initiation–to-response time	Interval from referral submission to specialist acknowledgment (min)	Platform time stamps	2-tailed *t* test or Mann-Whitney *U* test and multivariable regression
Change in bed use	Proportion of beds occupied at the receiving facilities	Platform dashboard+hospital records	Descriptive statistics and pretest-posttest comparison
Unanswered referral requests	Referrals without documented specialist response	Platform records	Frequency and rate calculation
Proportion of referrals answered within the urgency window	Percentage of referrals receiving a specialist response within predefined urgency-specific response targets	Platform time stamps	Frequencies, percentages, and chi-square test comparison
Referral acceptance and decline rate	Proportion of referrals accepted vs declined by the receiving facility	Platform records	Frequencies and chi-square test
Cases managed through remote advice	Referrals resolved via bidirectional specialist advice without transfer	Platform communication logs	Frequency and descriptive subgroup analysis
Patient satisfaction	Patient and attendant survey domain score	Structured survey (Multimedia Appendix 1)	Means and SDs and Likert distribution
Health care professional satisfaction and usability	Health care professional survey domain score	Structured survey (Multimedia Appendix 1)	Means and SDs and thematic integration

### Data Collection

Quantitative data will be collected automatically by the digital platform, recording referral time stamps, referral reason, urgency category, facility identifiers, bed availability status, and referral outcome. Preimplementation historical data will be extracted from paper registers at participating institutions. Health care professional surveys will be administered at 2 time points: within 2 weeks of deployment and at the end of the 6-month period. Patient surveys will be administered at the point of referral completion. Semistructured interviews will be conducted at midimplementation and at end of study. Focus group discussions will be held at approximately 3 months after deployment. Data quality will be checked through double-entry verification of a random 10% sample and monthly data monitoring committee reviews.

### Survey Instrument and Scoring

The structured survey instrument (Multimedia Appendix 1) comprises separate versions for health care providers and patients or attendants, covering usability, perceived efficiency, communication quality, referral safety, workload, and overall acceptability. Each domain contains 4 to 6 Likert-scale items (1=“strongly disagree”; 5=“strongly agree”). Domain scores are calculated as the mean of item responses.

### Interview and Focus Group Topic Guide

The semistructured interview and focus group discussion topic guide (Multimedia Appendix 2) covers 7 domains: platform usability, fit with clinical workflow, barriers to adoption, facilitators, perceived impact on referral decisions, bidirectional communication, and recommendations for scale-up.

### Data Analysis

Quantitative analysis will use SPSS (version 28; IBM Corp). Descriptive statistics, normality testing, pretest-posttest paired comparisons (2-tailed t test or Wilcoxon signed-rank test), chi-square or Fisher exact tests, and multivariable logistic regression will be conducted. A *P* value of less than .05 will be the significance threshold. Missing data will be handled using complete-case analysis (<10% missing) or multiple imputation (>10% missing). Historical referral data from participating institutions covering the 6 months immediately preceding platform deployment (December 2025-May 2026) will serve as the preimplementation comparison period.

Qualitative analysis will follow the six-step reflexive thematic analysis framework by Braun and Clarke [15]: (1) familiarization with the data, (2) generation of initial codes, (3) search for themes, (4) review of themes, (5) definition and naming of themes, and (6) production of the report. Interviews and focus group discussions will be audio recorded and transcribed verbatim prior to analysis. Two researchers will independently code the transcripts; coding disagreements will be resolved through discussion and consensus. Reflexive memos and an audit trail will be maintained throughout the analytical process. Recruitment will continue until thematic saturation is achieved.

Quantitative and qualitative datasets will first be analyzed independently, and results will subsequently be merged during the interpretation phase using a convergent mixed methods approach. Joint display matrices will be used to facilitate integration. Merged findings will be examined for convergence, complementarity, and divergence between the quantitative and qualitative strands.

### Ethical Considerations

Ethics approval has been obtained from the institutional review board of LRH, Peshawar (application reference: 399/LRH/MTI). All participants will provide written informed consent. All data will be deidentified prior to analysis. Data will be stored on encrypted institutional servers accessible only to named study personnel. No financial compensation will be provided to participants.

## Results

### Overview

This study received no external funding. As of manuscript submission, participant recruitment has not yet commenced because pilot deployment is scheduled to begin in June 2026. System development, stakeholder engagement, user-centered design, beta testing, and validation activities have been completed. Data collection is expected to continue through November 2026, quantitative and qualitative data analysis is planned for December 2026 through February 2027, and study findings are expected to be submitted for peer-reviewed publication in spring 2027. System development, user-centered iterative design, stakeholder engagement, beta testing, and validation have been completed. The pilot implementation phase is scheduled to commence in June 2026 across a phased sequence of participating facilities beginning with primary and secondary care hospitals and expanding to the 3 tertiary MTIs by July 2026. Data collection will continue for a 6-month period, with an anticipated sample of 5000 to 7000 emergency referral records. Qualitative data collection will begin at approximately 2 months following platform deployment and will continue until November 2026. Quantitative and qualitative data analysis is planned for December 2026 to February 2027.

### Study Timeline

The study timeline is outlined in Table 3.

**Table 3:** Study timeline.

Phase and activity	Time frame	Status
Phase 1: requirement gathering and stakeholder consultation	January 2026-February 2026	Completed
Phase 1: platform design, prototyping, and development	February 2026-May 2026	Completed
Phase 1: beta testing and iterative refinement	May 2026	Completed
Phase 1: validation testing and health care professional training	May 2026	Completed
Phase 2: pilot deployment and data collection—primary and secondary care facilities	June 2026-August 2026	Planned
Phase 2: pilot deployment and data collection—tertiary MTIs^a^	July 2026-November 2026	Planned
Phase 2: qualitative data collection (surveys, interviews, and focus groups)	August 2026-November 2026	Planned
Phase 3: data analysis (quantitative and qualitative)	December 2026-February 2027	Planned
Phase 3: report preparation and policy dissemination	March 2027-April 2027	Planned

^a^MTI: Medical Teaching Institution.

## Discussion

### Principal Findings

This protocol describes the design, development, and planned evaluation of a neurosurgery-led digital emergency referral system in KP, Pakistan. It is anticipated that the study will show that a structured mobile- and web-based referral platform may decrease the rate of unnecessary emergency transfers, decrease the time spent on initiating a referral and responding to it, and enhance bed occupancy rates in tertiary care facilities.

### Comparison With Prior Work

This research is based on the accumulating evidence on digital referral systems in LMICs, such as the Mobile Obstetric Referral Emergency System in Liberia [8], the Saudi Medical Appointments and Referrals Centre in Saudi Arabia [9], web-based neurosurgical referral systems in higher-income countries [10], and evidence from Indonesia [11] and systematic reviews on e-referral solutions [12].

### Strengths and Limitations

The strengths are a multicenter design with all levels of health care, high expected volume of samples, and the combination of quantitative and qualitative approaches. Limitations are the lack of randomization, the digital literacy and connectivity differences between facilities, the possible incompleteness of preimplementation paper records, and operational pressures that can influence study compliance.

### Future Directions

Future directions could include provincial scale-up, interoperability with the National Database and Registration Authority in Pakistan, artificial intelligence–assisted triage, and economic assessment.

The results of the study will be shared with the KP Health Department and the MTI administrations through peer-reviewed publications, conference presentations, and formal reports. Open access publication will be given priority.

### Conclusions

This protocol describes a stringent mixed methods implementation study of a neurosurgery-based digital emergency referral platform in KP, Pakistan. The results can be used to offer a transferable evidence base to justify the use of digital referral systems in KP and similar LMIC contexts.

## Data Availability

Deidentified data generated during this study may be made available from the corresponding author on reasonable request subject to institutional approvals, ethics approval conditions, and applicable data protection requirements.

## Appendix

Multimedia Appendix 1 Health care provider and patient survey instruments for the evaluation of the KP MTI referral app.

Multimedia Appendix 2 Semistructured interview and focus group discussion topic guides.

## Abbreviations

HMC: Hayatabad Medical Complex
KP: Khyber Pakhtunkhwa
KTH: Khyber Teaching Hospital
LMIC: low- and middle-income country
LRH: Lady Reading Hospital
MTI: Medical Teaching Institution
