# Supplementary material for: Neurosurgery-Led Digital Emergency Referral System in Khyber Pakhtunkhwa, Pakistan: Protocol for a Mixed Methods Implementation Study
Source: JMIR Res Protoc. 2026 Jul 17;15:e90331. doi: 10.2196/90331 (PMC13428199; doi:10.2196/90331)
Supplement: Multimedia Appendix 1 [file resprot_v15i1e90331_app1.docx]

**Multimedia Appendix 1**

**Provider and Patient Survey Instrument with Scoring Plan**

This appendix contains the structured survey instruments for healthcare providers and patients or attendants, as described in the Methods section of the main manuscript. Surveys assess usability, satisfaction, perceived efficiency, communication quality, workload, referral safety, and overall acceptability of the digital emergency referral platform. All items are rated on a five-point Likert scale (1 = Strongly Disagree; 5 = Strongly Agree) unless otherwise indicated. Surveys are available in English, Urdu, and Pashto; research assistants will read items aloud for participants with low literacy.

# Part A: Healthcare Provider Survey

Instructions: Please rate each statement according to your experience with the KP MTI Referral Application. Circle or mark the number that best represents your opinion. There are no right or wrong answers. Your responses are anonymous.

Participant Role (please tick): □ Emergency Physician □ Neurosurgical Consultant □ Medical Officer □ Nurse □ Bed Manager □ Administrative Staff □ Other: _______

Facility Level (please tick): □ Tehsil (subdistrict) Hospital □ District Hospital □ Medical Teaching Institution (MTI)

## Domain 1: Platform Usability

| **Item** | **1 Strongly Disagree** | **2 Disagree** | **3 Neutral** | **4 Agree** | **5 Strongly Agree** |
| --- | --- | --- | --- | --- | --- |
| 1.1 The referral form is easy to navigate on the mobile device. |  |  |  |  |  |
| 1.2 The steps to complete and submit a referral are clearly presented. |  |  |  |  |  |
| 1.3 Notification alerts are clear and easy to understand. |  |  |  |  |  |
| 1.4 The platform interface is visually clear and uncluttered. |  |  |  |  |  |
| 1.5 I can complete a referral efficiently without requiring additional help. |  |  |  |  |  |

## Domain 2: Perceived Efficiency

| **Item** | **1 Strongly Disagree** | **2 Disagree** | **3 Neutral** | **4 Agree** | **5 Strongly Agree** |
| --- | --- | --- | --- | --- | --- |
| 2.1 Using this platform makes the referral process faster. |  |  |  |  |  |
| 2.2 The platform fits well into my existing clinical workflow. |  |  |  |  |  |
| 2.3 Real-time bed availability information helps me make better referral decisions. |  |  |  |  |  |
| 2.4 The time I spend on referral documentation has changed since using the platform. |  |  |  |  |  |
| 2.5 The platform reduces unnecessary patient transfers in my experience. |  |  |  |  |  |

## Domain 3: Communication Quality

| **Item** | **1 Strongly Disagree** | **2 Disagree** | **3 Neutral** | **4 Agree** | **5 Strongly Agree** |
| --- | --- | --- | --- | --- | --- |
| 3.1 The platform improves communication between referring and receiving facilities. |  |  |  |  |  |
| 3.2 Receiving specialists respond to referrals in a timely manner through the platform. |  |  |  |  |  |
| 3.3 The bidirectional messaging function is useful for clinical decision-making. |  |  |  |  |  |
| 3.4 I am able to provide or receive management advice without requiring patient transfer when appropriate. |  |  |  |  |  |
| 3.5 The urgency classification of referrals is clearly communicated through the platform. |  |  |  |  |  |

## Domain 4: Referral Safety

| **Item** | **1 Strongly Disagree** | **2 Disagree** | **3 Neutral** | **4 Agree** | **5 Strongly Agree** |
| --- | --- | --- | --- | --- | --- |
| 4.1 The platform helps ensure that important clinical information is communicated during referral. |  |  |  |  |  |
| 4.2 I feel confident that referrals made through this platform reach the correct specialist. |  |  |  |  |  |
| 4.3 The platform helps reduce the risk of a patient being referred to a facility without available capacity. |  |  |  |  |  |
| 4.4 The urgency-based triage categories reflect real clinical prioritization needs. |  |  |  |  |  |

## Domain 5: Workload

| **Item** | **1 Strongly Disagree** | **2 Disagree** | **3 Neutral** | **4 Agree** | **5 Strongly Agree** |
| --- | --- | --- | --- | --- | --- |
| 5.1 The platform has reduced my administrative burden compared to paper-based referral. |  |  |  |  |  |
| 5.2 Completing referral forms on the platform takes a reasonable amount of time. |  |  |  |  |  |
| 5.3 Responding to incoming referrals through the platform is manageable within my workload. |  |  |  |  |  |
| 5.4 I do not experience excessive notification alerts from the platform during my shift. |  |  |  |  |  |

## Domain 6: Overall Acceptability

| **Item** | **1 Strongly Disagree** | **2 Disagree** | **3 Neutral** | **4 Agree** | **5 Strongly Agree** |
| --- | --- | --- | --- | --- | --- |
| 6.1 Overall, I am satisfied with the KP MTI Referral Application. |  |  |  |  |  |
| 6.2 I would recommend this platform to colleagues in other facilities. |  |  |  |  |  |
| 6.3 I intend to continue using this platform for emergency referrals. |  |  |  |  |  |
| 6.4 I believe this platform should be expanded to all hospitals in Khyber Pakhtunkhwa. |  |  |  |  |  |
| 6.5 The training I received was sufficient to use the platform confidently. |  |  |  |  |  |

## Open-Ended Questions (Provider Survey)

P1. What features of the platform do you find most useful? (Please describe)

_________________________________________________________________________________

P2. What challenges or barriers have you encountered when using the platform?

_________________________________________________________________________________

P3. What improvements would you suggest to make the platform more effective in your setting?

_________________________________________________________________________________

## Scoring Plan – Provider Survey

Each domain score is calculated as the mean of the item responses within that domain (range 1 to 5). Higher scores indicate greater usability, satisfaction, efficiency, or acceptability, as appropriate to each domain. A composite Total Acceptability and Usability Score is derived as the mean of all six domain scores. Domain and total scores will be reported as means and standard deviations. Pre- and post-implementation comparisons will use paired t-tests or Wilcoxon signed-rank tests as appropriate to data distribution.

| **Domain** | **Items** | **Number of Items** | **Score Range** |
| --- | --- | --- | --- |
| Usability | 1.1–1.5 | 5 | 1–5 |
| Perceived Efficiency | 2.1–2.5 | 5 | 1–5 |
| Communication Quality | 3.1–3.5 | 5 | 1–5 |
| Referral Safety | 4.1–4.4 | 4 | 1–5 |
| Workload | 5.1–5.4 | 4 | 1–5 |
| Overall Acceptability | 6.1–6.5 | 5 | 1–5 |
| **TOTAL (composite mean)** | All items | 28 | 1–5 |

# Part B: Patient and Attendant Survey

Instructions: This survey asks about your experience with the referral process during your hospital visit. A research assistant will help you complete this form if needed. Your answers are confidential and will not affect your care.

Respondent (please tick): □ Patient □ Attendant/Next of kin

## Domain 1: Satisfaction with the Referral Process

| **Item** | **1 Strongly Disagree** | **2 Disagree** | **3 Neutral** | **4 Agree** | **5 Strongly Agree** |
| --- | --- | --- | --- | --- | --- |
| PA1. I was informed about the referral process before I was transferred. |  |  |  |  |  |
| PA2. The referral was completed in a reasonable amount of time. |  |  |  |  |  |
| PA3. I was satisfied with the way staff handled my referral. |  |  |  |  |  |
| PA4. The receiving hospital was informed about my condition before I arrived. |  |  |  |  |  |

## Domain 2: Perceived Communication during Transfer

| **Item** | **1 Strongly Disagree** | **2 Disagree** | **3 Neutral** | **4 Agree** | **5 Strongly Agree** |
| --- | --- | --- | --- | --- | --- |
| PA5. Staff explained the reason for my referral clearly. |  |  |  |  |  |
| PA6. I felt that important information about my health was passed to the receiving hospital. |  |  |  |  |  |
| PA7. I was able to ask questions about the referral process. |  |  |  |  |  |

## Domain 3: Acceptability of the Digital Process

| **Item** | **1 Strongly Disagree** | **2 Disagree** | **3 Neutral** | **4 Agree** | **5 Strongly Agree** |
| --- | --- | --- | --- | --- | --- |
| PA8. I am comfortable with staff using a digital system to manage my referral. |  |  |  |  |  |
| PA9. I believe the digital referral system helped make my transfer safer. |  |  |  |  |  |
| PA10. Overall, I am satisfied with the care I received during the referral process. |  |  |  |  |  |

## Open-Ended Question (Patient and Attendant Survey)

Q1. Is there anything you would like to share about your referral experience?

_________________________________________________________________________________

## Scoring Plan – Patient and Attendant Survey

Each domain score is calculated as the mean of item responses within that domain (range 1 to 5). A composite patient satisfaction score is derived as the mean of all ten item responses. Domain and composite scores will be reported as means and standard deviations.

| **Domain** | **Items** | **Number of Items** | **Score Range** |
| --- | --- | --- | --- |
| Satisfaction with referral process | PA1–PA4 | 4 | 1–5 |
| Perceived communication | PA5–PA7 | 3 | 1–5 |
| Acceptability of digital process | PA8–PA10 | 3 | 1–5 |
| **TOTAL (composite mean)** | All items | 10 | 1–5 |
