# Supplementary material for: Neurosurgery-Led Digital Emergency Referral System in Khyber Pakhtunkhwa, Pakistan: Protocol for a Mixed Methods Implementation Study
Source: JMIR Res Protoc. 2026 Jul 17;15:e90331. doi: 10.2196/90331 (PMC13428199; doi:10.2196/90331)
Supplement: Multimedia Appendix 2 [file resprot_v15i1e90331_app2.docx]

**Multimedia Appendix 2**

**Semi-Structured Interview and Focus Group Discussion Guide**

This appendix contains the semi-structured interview and focus group discussion guides used for the qualitative component of the study evaluating the KP MTI Referral Application. The guide was developed to explore healthcare provider and patient experiences, perceived usability, workflow integration, communication quality, barriers and facilitators to implementation, and recommendations for future scale-up of the digital emergency referral platform.

Interviews and focus group discussions will be conducted by trained research personnel in English, Urdu, or Pashto according to participant preference. Interviews are expected to last approximately 20–40 minutes, while focus group discussions are expected to last 45–60 minutes. With participant permission, sessions will be audio-recorded and transcribed for thematic analysis.

**Part A: Healthcare Provider Interview and Focus Group Guide**

**Introduction Script**

Thank you for participating in this interview/discussion. We are conducting this study to understand your experience using the KP MTI Referral Application for emergency referrals. Your responses will help us evaluate the system and identify areas for improvement. There are no right or wrong answers. We are interested in your honest opinions and experiences. Your participation is voluntary, and your responses will remain confidential. You may decline to answer any question or stop the interview at any time.

**Participant Information**

Participant role:

- Emergency Physician
- Neurosurgical Consultant
- Medical Officer
- Nurse
- Bed Manager
- Administrative Staff
- Other: __________

Facility level:

- Tehsil (subdistrict-level) Hospital
- District Headquarters Hospital
- Medical Teaching Institution (MTI)

Duration of platform use:

- Less than 1 month
- 1–3 months
- More than 3 months

**Domain 1: Platform Usability**

Core Questions:

1. Can you describe your overall experience using the KP MTI Referral Application?
2. How easy or difficult is the platform to use during routine clinical work?
3. Which features of the platform do you find most useful?

Probes:

- Referral form completion
- Navigation through the application
- Notification system
- Uploading images/documents
- Tracking referrals
- Mobile versus desktop usability

**Domain 2: Integration With Clinical Workflow**

Core Questions:
4. How well does the platform fit into your existing referral workflow?
5. Has the platform changed the way referrals are managed in your department or facility?

Probes:

- Time required for referral submission
- Communication with receiving hospitals
- Bed availability checks
- Documentation burden
- Workflow interruptions

**Domain 3: Communication and Clinical Decision-Making**

Core Questions:
6. How has the platform affected communication between referring and receiving facilities?
7. What has been your experience with the bidirectional communication feature?

Probes:

- Timeliness of specialist responses
- Ability to obtain remote management advice
- Reduction in unnecessary transfers
- Clarity of communication
- Referral acknowledgment process

**Domain 4: Urgency Classification and Referral Prioritization**

Core Questions:
8. What are your views on the urgency-based triage categories (emergency, urgent, routine)?
9. Do you think the urgency classification system reflects real clinical priorities?

Probes:

- Response times
- Automated notifications
- Alert burden/fatigue
- Prioritization during busy periods
- Suggestions for improvement

**Domain 5: Barriers to Adoption**

Core Questions:
10. What challenges or barriers have you encountered while using the platform?
11. Were there any difficulties during implementation or training?

Probes:

- Internet connectivity
- Technical issues
- Digital literacy
- Staffing constraints
- Resistance to change
- Availability of devices
- Workload concerns

**Domain 6: Facilitators and Positive Experiences**

Core Questions:
12. What factors have helped successful implementation of the platform in your facility?
13. What aspects of the system encourage continued use?

Probes:

- Training quality
- Administrative support
- Ease of use
- Perceived clinical benefit
- Improved coordination
- Reduced delays

**Domain 7: Perceived Impact on Patient Care**

Core Questions:
14. In your opinion, how has the platform affected patient care and referral safety?
15. Have you observed any changes in unnecessary referrals or transfer delays?

Probes:

- Access to specialist input
- Bed utilization
- Referral tracking
- Patient outcomes
- Emergency care coordination

**Domain 8: Recommendations and Future Scale-Up**

Core Questions:
16. What improvements would you recommend for the platform?
17. Do you believe this system should be expanded to other hospitals or specialties?

Probes:

- Additional features
- Training recommendations
- Technical improvements
- Provincial/national integration
- Artificial intelligence-assisted triage
- Sustainability considerations

**Closing Question**

1. Is there anything else you would like to share about your experience with the digital referral system?

**Part B: Patient and Attendant Interview Guide**

**Introduction Script**

We would like to ask about your experience with the hospital referral process. Your responses will help improve referral services and patient care. Participation is voluntary, and your answers will remain confidential.

**Participant Information**

Respondent:

- Patient
- Attendant/Next of kin

Referred from:

- Primary care facility
- District hospital
- Medical Teaching Institution

**Domain 1: Understanding of the Referral Process**

Core Questions:

1. Were you informed about why the referral was needed?
2. Did healthcare staff explain the referral process clearly?

Probes:

- Information provided before transfer
- Understanding of destination hospital
- Opportunity to ask questions

**Domain 2: Experience During Referral**

Core Questions:
3. How would you describe your experience during the referral process?
4. Did you experience any delays or difficulties?

Probes:

- Waiting time
- Coordination between hospitals
- Transportation issues
- Staff communication

**Domain 3: Perceptions of the Digital Referral System**

Core Questions:
5. What do you think about healthcare staff using a digital system to manage referrals?
6. Do you think the digital system improved your referral experience?

Probes:

- Confidence in referral coordination
- Perceived safety
- Faster communication
- Preparedness of receiving hospital

**Domain 4: Overall Satisfaction and Recommendations**

Core Questions:
7. Overall, how satisfied were you with the referral process?
8. What improvements would you suggest?

Probes:

- Communication
- Waiting times
- Staff behavior
- Referral coordination

**Closing Question**

1. Is there anything else you would like to share about your referral experience?
